# Evaluation of the ocular surface mycobiota in clinically normal horses

**DOI:** 10.1371/journal.pone.0246537

**Published:** 2021-02-04

**Authors:** Mary L. Walsh, Courtney Meason-Smith, Carolyn Arnold, Jan S. Suchodolski, Erin M. Scott

**Affiliations:** 1 Department of Small Animal Clinical Sciences, College of Veterinary Medicine & Biomedical Sciences, Texas A&M University, College Station, Texas, United States of America; 2 Department of Large Animal Clinical Sciences, College of Veterinary Medicine & Biomedical Sciences, Texas A&M University, College Station, Texas, United States of America; Westen University of Health Sciences, UNITED STATES

## Abstract

The eye is host to myriad bacterial, fungal, and viral organisms that likely influence ocular surface physiology in normal and diseased states. The ocular surface mycobiota of horses has not yet been described using NGS techniques. This study aimed to characterize the ocular surface fungal microbiota (mycobiota) in healthy horses in 2 environmental conditions (stalled versus pasture). Conjunctival swabs of both eyes were obtained from 7 adult stallions stabled in an open-air pavilion and 5 adult mares living on pasture. Genomic DNA was extracted from ocular surface swabs and sequenced using primers that target the Internal Transcribed Spacer 1 (ITS1) region of the fungal genome on an Illumina platform. Sequences were processed using Quantitative Insights Into Molecular Ecology (QIIME 2.0) and taxonomy assigned with the Findley et al. 2013 ITS1 database. The most abundant genera identified were *Leptosphaerulina* (22.7%), *unclassified Pleosporaceae* (17.3%), *Cladosporium* (16.2%), *Alternaria* (9.8%), *unclassified Pleosporales* (4.4%), *unclassified Montagnulaceae* (2.9%), *Fusarium* (2.5%), and *Pestalotiopsis* (1.4%). Fungal community composition (Jaccard, R = 0.460, p = 0.001) and structure (Bray-Curtis, R = 0.811, p = 0.001) were significantly different between pastured mares and stabled stallions. The ocular surface of pastured mares had significantly increased fungal species richness and diversity compared to stabled stallions (Shannon p = 0.0224, Chao1 p = 0.0118, Observed OTUs p = 0.0241). Relative abundances of *Aspergillus* (p = 0.005) and *Alternaria* spp. (p = 0.002) were significantly increased in the mycobiota of pastured mares. This is the first report to describe the mycobiota of the equine ocular surface. Environmental factors such as housing influence the composition, structure, and richness of the equine ocular surface mycobiota.

## Introduction

The horse is a unique animal model for fungal keratitis as equids develop this vision-threatening disease with more frequency than other companion animals [1]. Horses have large, prominent globes which increase environmental exposure while also predisposing the eye to traumatic injury. Ocular trauma often creates a break or defect in the corneal epithelium, allowing for commensal or opportunistic fungi to colonize the exposed corneal stroma which may result in pathogenicity [2–4]. Culture based diagnostic techniques are used frequently in clinical veterinary practice to determine the specific organism causing disease and to target treatment. Cultures of affected equine eyes have predominantly isolated *Aspergillus*, *Fusarium*, *Penicillium*, and *Cladosporium* spp., but are variable in regards to geographic environment and season [4–8].

Previous studies indicate that fungal species may be a commensal population on the equine ocular surface, with 13–95% of the swabs from healthy horse eyes positive for fungal growth [1, 9–17]. It has been hypothesized that there may be a transient nature to the conjunctival microbiome as an effect of the environment [3]. Johns et al. identified only 13% of conjunctival swabs from healthy horses with positive fungal growth in the United Kingdom; however, this is also an area with a low prevalence of fungal keratitis [13]. In contrast, a study in Iran identified 77% of ocular samples from healthy horses had positive growth with *Aspergillus*, *Rhizopus* and *Penicillium* isolated most frequently [14]. Physician ophthalmology studies have identified similar risk factors in people where trauma is a predisposing factor to developing fungal keratitis in 94% of patients [18], and there is a higher prevalence in warmer climates such as South Asia [18, 19].

Next generation sequencing (NGS) techniques are able to detect a diverse population of microorganisms than previously described using culture-based techniques. With regard to the bacterial ocular surface microbiome, culture-independent molecular-based methods, such as 16S rRNA gene sequencing, identified unexpectedly diverse and stable bacterial communities occupying the ocular surface of humans [20–24], horses [25], cats [26] and dogs [27, 28]. The surface fungal microbiome has been less extensively studied. Findlay et al. used NGS to define the fungal microbiome across multiple epidermal surfaces in humans, demonstrating that the mycobiota is dependent on body site location [29]. More recently, NGS studies evaluating the human ocular surface mycobiome using ITS1 or ITS2 sequencing have identified a greater degree of diversity compared to conventional cultivable methods, including several fungal genera associated with the healthy human conjunctiva [30, 31]. In veterinary medicine, evaluation of the surface fungal microbiome of the body is limited to the skin and conjunctiva of cats [32, 33] and dogs [34], where NGS has described the presence of several organisms which are identifiable by traditional culture techniques.

Presently, there are no published reports evaluating the ocular surface mycobiota of horses using molecular-based techniques. Furthermore, the characterization of a resident mycobiome in healthy subjects will allow for changes associated with a specific disease processes, such as fungal keratitis, to improve treatment therapy and outcomes. The purpose of this study is to describe the ocular surface fungal microbiota (mycobiota) in healthy horses, and to evaluate the effect of housing conditions on the equine ocular surface mycobiota.

## Materials and methods

### Participants

The study was approved by the Texas A&M University Institutional Animal Care and Use Committee (Animal Use Protocol #2017–0333). Twelve horses, free of ocular disease, were selected from the teaching herd at the Department of Large Animal Clinical Sciences at Texas A&M University College of Veterinary Medicine & Biomedical Sciences. The ocular surface microbiome was previously described from the same herd of horses [25]. The horses ranged in age from 7–25 years old and included five mares and seven stallions. Represented breeds included: Quarter horse (n = 9), Thoroughbred (n = 1), Morgan-cross (n = 1) and an Arabian (n = 1). The study was performed in December in east-central Texas, and during the sampling period the temperature was 56 degrees F with an average humidity of 70%. The mares were pastured, whereas the stallions were housed year-round in individual stalls within an open-air pavilion. Horses were provided with free-choice water and hay, and were fed grain daily.

### Sample collection

All horses had a complete ophthalmic examination performed by a board-certified veterinary ophthalmologist (EMS). This included evaluation of the anterior segment of the eye by slit-lamp biomicroscopy (SL-17, Kowa Optimed Inc., Torrance, CA), and the posterior segment of the eye by indirect ophthalmoscopy (Vantage Plus Wireless Headset, Keeler Instruments Inc., Malvern, PA), as previously described [25]. A routine minimal ophthalmic database was also performed including Schirmer tear test measurements (Intervet Inc., Summit, NJ), fluorescein staining (Amcon Laboratories Inc., St. Louis, MO), and tonometry (Tono-Pen, Dan Scott and Associates, Inc., Westerville, OH). Any horse with an abnormal ophthalmic exam or minimal database result was excluded from the study.

Conjunctival swab samples were collected after the Schirmer tear test and before fluorescein staining and tonometry in order to prevent contamination or dilution of the sample. A volume of 0.2 ml 0.5% tetracaine (Bausch & Lomb Inc., Tampa, FL) was placed on the ocular surface of each eye to provide topical analgesia, and allow for deep swabbing with applied pressure. The inferior conjunctival fornix of both eyes was sampled with Isohelix buccal swabs (Boca Scientific, Inc. Westwood, MA). Two swabs were used for each site, and each side of the swab was rubbed in the conjunctival fornix 10 times, as previously described [25]. The swabs were collected in DNeasy Powerbead tubes with 750-μl buffer containing guanidine thiocyanate (QIAGEN, Inc., Germantown, MD). A volume of 0.2 ml 0.5% tetracaine was placed on an unused swab at the same time and place of subject testing to serve as a negative control to confirm a lack of environmental contamination. All samples were stored at -80 degrees C for ten months until the extractions were performed.

### DNA extraction and sequencing

Genomic DNA was extracted from the swabs and negative control using the DNeasy Powersoil DNA isolation kit (QIAGEN, Inc., Germantown, MD) following the manufacturer’s instructions.

### ITS sequencing and sequence analysis

Illumina sequencing (Illumina Inc.; San Diego CA, USA) of all samples was performed on an Illumina MiSeq instrument at MR DNA Laboratory (www.mrdnalab.com, Shallowater, TX, USA) using ITS1F (50- CTTGGTCATTTAGA GGAAGTAA-30) and ITS2R (50- GCTGCGTTCTTCATCGATGC-30) primers that amplified the internal transcribed spacer (ITS-1) region, a noncoding segment of genome found within the ribosomal genes of all eukaryotes [35]. Primers were used in a 35 cycle PCR using the HotStarTaq Plus Master Mix Kit (QIAGEN, Inc., Germantown, MD) under the following conditions: 94°C for 3 minutes, followed by 30–35 cycles of 94°C for 30 seconds, 53°C for 40 seconds and 72°C for 1 minute, after which a final elongation step at 72°C for 5 minutes was performed. After amplification, PCR products were checked in 2% agarose gel to determine the success of amplification and the relative intensity of bands. Multiple samples were pooled together (e.g., 100 samples) in equal proportions based on their molecular weight and DNA concentrations. Pooled samples were purified using calibrated Ampure XP beads. Then the pooled and purified PCR product was used to prepare Illumina Truseq nano DNA library.

Sequences from only the forward reads were then processed in the open-source bioinformatics software Quantitative Insights into Microbial Ecology, QIIME-2 [36]. Quality filtering was performed and operational taxonomic units (OTUs; group of similar sequences that represents a taxonomic unit of a fungal species or genus) generated using the closed reference picking command and the ITS sequence database [29]. Taxonomic assignments were made with a formatted version of the ITS taxonomy file [29]. OTU tables were rarefied at 12,960 sequences for diversity analyses only. Alpha diversity was measured using Chao1, observed OTUs, and Shannon metrics. Beta diversity was measured using weighted Jaccard and Bray Curtis metrics. These calculations were performed for each possible pair of samples and the distance matrix generated was then used to create 3D PCoA plots.

### Statistical analysis

The relative abundance tables were combined for all taxonomic levels (Phylum, Class, Order, Family and Genus) and filtered to include taxa present at greater than 1%. Using the statistical software JMP Pro 14 (SAS Institute, Inc.; Cary, NC, USA), data were tested for normality with a Shapiro-Wilk test. Non-parametric tests were performed on data due to lack of normal distribution. A Wilcoxon signed rank test (Kruskal-Wallis) was performed to determine whether the mean value (relative abundance or alpha diversity) was significantly different between groups (horse, environment) (p < 0.05). When significant, a Wilcoxon signed rank multiple comparisons test (Mann-Whitney U) was performed to identify the horse(s) with significant differences. To test for differences in the beta diversity of samples by horse, eye, or environment, the analysis of similarities (ANOSIM) function in the statistical software PRIMER 6 (PRIMER-E Ltd.; Luton, UK) was performed on the distance matrices generated in QIIME using the Jaccard and Bray Curtis metrics. R values were calculated for each pair-wise comparison between groups and a global R statistic was calculated for the factor under study (horse, environment) (ANOSIM, PRIMER 6). The combined and filtered relative abundance tables were also used in linear discriminant analysis (LDA) effect size (LEfSe) [37] to determine significant differences between horses, or environment. All p-values were corrected for multiple comparisons using the Benjamini and Hochberg false discovery rate [38].

## Results

### Sequence analysis

The negative control sample consisting of an unused swab and 0.2 ml 0.5% tetracaine for detection of DNA contamination was negative on PCR amplification, indicating sample and DNA extraction processes did not contain contaminants. This sample control was also included in sequencing and verified to contain < 1% of total OTUs for all fungal taxa. All 24 samples collected from both eyes of each horse at one timepoint were positive for PCR amplification and sent for sequencing. A total of 2,750,499 sequences were amplified (Min 12,929; Max 118,600; Median 72,209; Mean 66,849; SD 27,171). Following quality filtering and removal of chimeras, 445,207 sequences were identified and used for data analysis. The relative abundance of each fungal taxon was defined for each individual sample. Data were deposited in the National Center for Biotechnology Information (NCBI) Sequence Read Archive (SRA) under the accession number PRJNA659281.

### Microbial community structure

**Fig 1:**
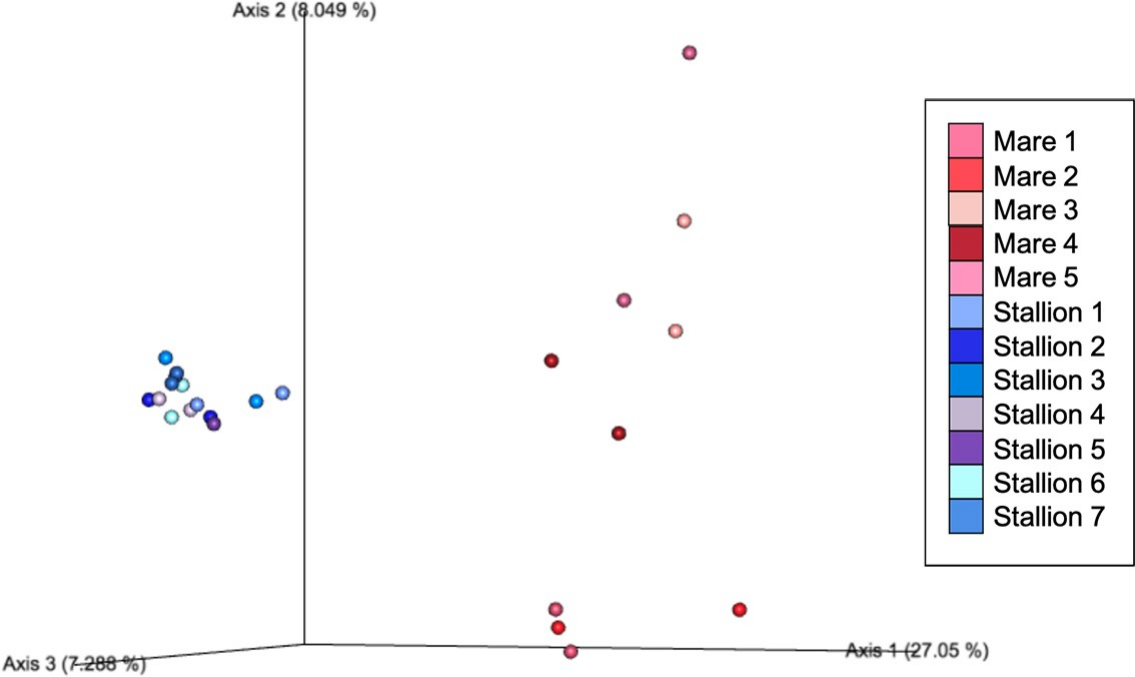
Principal coordinate analysis plot of Bray-Curtis distance matrices between each eye of 12 horses. Each dot represents the microbial composition of one eye. The dots which are shades of blue representing stabled stallions are clustered, indicating a similar community structure within that housing environment. The dots which are shades of red representing pastured mares are more dispersed, indicating that their individual mycobiome structures are more diverse, and distinct from stabled stallions.

Beta diversity measures were analyzed to examine taxonomic diversity between samples. Bray-Curtis and Jaccard distance matrices demonstrated that individual horses had a significant effect on the community structure of their mycobiome based on ANOSIM (R = 0.39, p = 0.001; R = 0.32, p = 0.002, respectively). Principle coordinate analysis plots demonstrate that, for many horses, both eyes of the same horse had a similar mycobiome composition (Fig 1). Clustering is observed more substantially among stallions 1–7 as a result of having a similar mycobiome composition within the group; whereas, it is less notable among mares 1–5. Additionally, the horse’s environment had a significant effect on beta diversity based on ANOSIM (Bray-Curtis R = 0.81, p = 0.001; Jaccard R = 0.46, p = 0.001), as illustrated by clustering of the eyes of stabled stallions on principal coordinate analysis plots compared to pastured mares (Fig 1). Minimal clustering was seen among mares living on pasture indicating that their ocular surface had a more taxonomically diverse mycobiome compared to stabled stallions (Fig 1).

### Species richness and diversity

**Fig 2:**
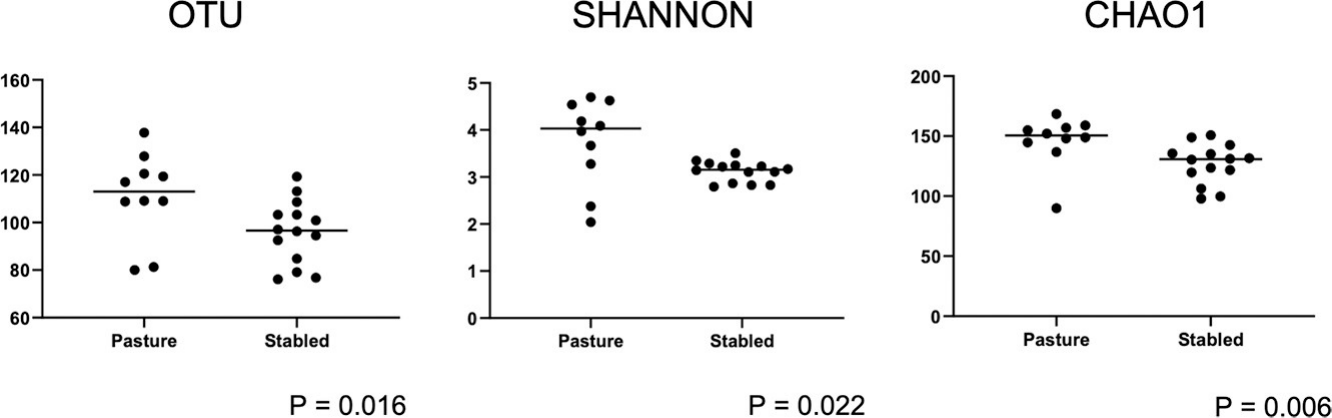
Scatterplots and statistical evaluation of ITSI sequences from 12 healthy horses housed either in an outdoor pavilion (stabled stallions) or on pasture (pastured mares). Each dot corresponds to one of 24 eyes from 12 healthy horses. Pastured mares have a significantly more diverse ocular mycobiome compared to stabled stallions (Wilcoxon signed rank; OTU p = 0.016, Shannon p = 0.022, Chao1 p = 0.006).

Alpha diversity measures from samples collected from the 12 horses were analyzed and compared between individual horses and environments. Species richness (observed OTUs and Chao1) and evenness of diversity (Shannon) were analyzed to examine taxonomic diversity within a sample. There was no significant difference between individual horses for all three alpha diversity matrices (p > 0.05) (S1 Table). There were significant differences in species richness (observed OTUs, p = 0.016; Chao1, p = 0.006) and evenness (Shannon, p = 0.022) between the two different housing environments (S1 Table and Fig 2). Mares living on pasture had greater species richness and diversity when compared to stallions stabled in an open-air pavilion (Fig 2).

### Microbial community composition

**Fig 3:**
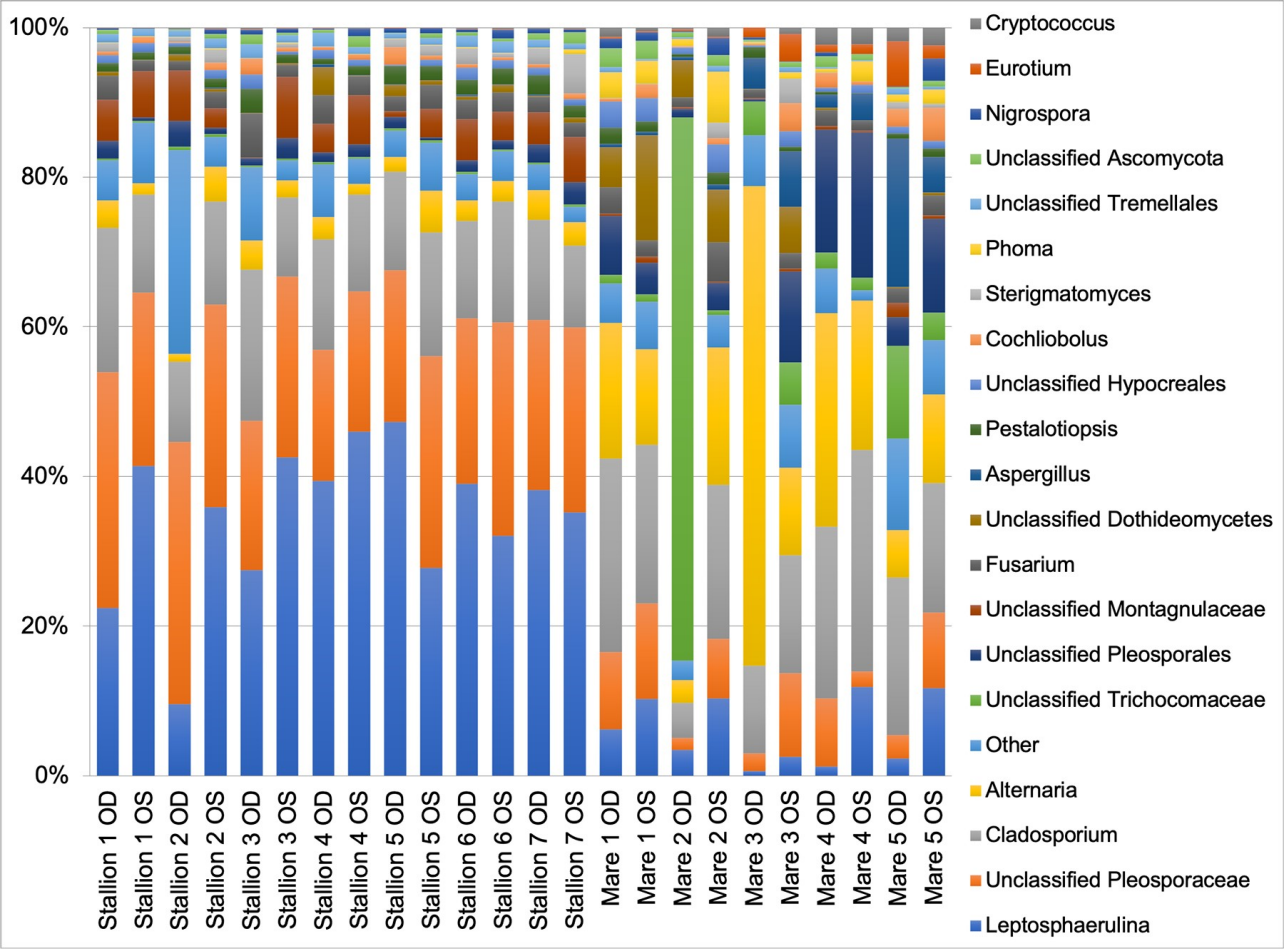
Composition of the ocular surface mycobiome in healthy horses. Relative abundance of taxa at the genus level. Each bar represents the left (OS) or right (OD) eyes of 12 horses.

Data from all 24 eyes were averaged to describe the prominent fungal genera inhabiting the healthy equine ocular surface. Table 1 summarizes the mean relative abundance of fungal genera identified. Two phyla were detected: Ascomycota and Basidiomycota. Ascomycota represented 95% of the organisms identified and Basidiomycota represented 5%. Within Ascomycota, the following classes were identified: Dothideomycetes, Eurotiomycetes and Sordariomycetes. Within Phylum Basidiomycota, classes Agaricostilbomycetes and Tremellomycetes were represented. A total of 21 genera were present at greater than 1% relative abundance in at least one sample. The most abundant genera were *Leptosphaerulina* (22.7%), *unclassified Pleosporaceae* (17.3%), *Cladosporium* (16.2%) and *Alternaria* (9.8%) (Fig 3). When compared to previous culture-based studies, the fungal taxa previously thought to account for the majority of the core ocular surface mycobiome in horses only represented a small proportion of all the fungal genera sequenced in this study. As shown in Table 1, two frequently cultured fungi, *Aspergillus* and *Fusarium* species, had a mean relative abundance of 1.9% and 2.5%, respectively. Individual variation in relative abundances of fungal taxa was observed both between eyes and between horses (Fig 3). Throughout all samples, an average of 102 different OTUs were sequenced.

**Table 1:** Taxa present at ≥1% mean relative abundance in at least one healthy horse eye.

TAXON Class	Mean Relative Abundance (%)	Standard Deviation (%)	Number of eyes with positive detection (n = 24)
*Genus*			
**Dothideomycetes**			
*Leptosphaerulina*	22.7	16.5	23
*Unclassified Pleosporaceae*	17.3	10	24
*Cladosporium*	16.2	5.6	24
*Alternaria*	9.8	13.6	24
*Unclassified Pleosporales*	4.4	5.3	19
*Unclassified Montagnulaceae*	2.9	2.7	13
*Unclassified Dothideomycetes*	2.0	3.4	8
*Cochliobolus*	1.2	1.2	7
*Phoma*	0.94	1.6	5
**Eurotiomycetes**			
*Unclassified Trichocomaceae*	4.5	14.7	9
*Aspergillus*	1.9	4.3	6
*Eurotium*	0.67	1.4	5
**Sordariomycetes**			
*Fusarium*	2.5	1.2	24
*Pestalotiopsis*	1.4	0.77	16
*Unclassified Hypocreales*	1.3	0.97	12
*Nigrospora*	0.68	0.73	6
*Unclassified Ascomycota*	0.83	0.69	7
**Agaricostilbomycetes**			
*Sterigmatomyces*	1.0	1.3	9
**Tremellomycetes**			
*Unclassified Tremellales*	0.87	0.5	9
*Cryptococcus*	0.60	0.8	6
*Other*	6.2	5.2	24

Mean relative percentages and standard deviation of the most abundant fungal genera, annotated to the level of genus based on sequencing of the ITS1 region are shown.

**Fig 4:**
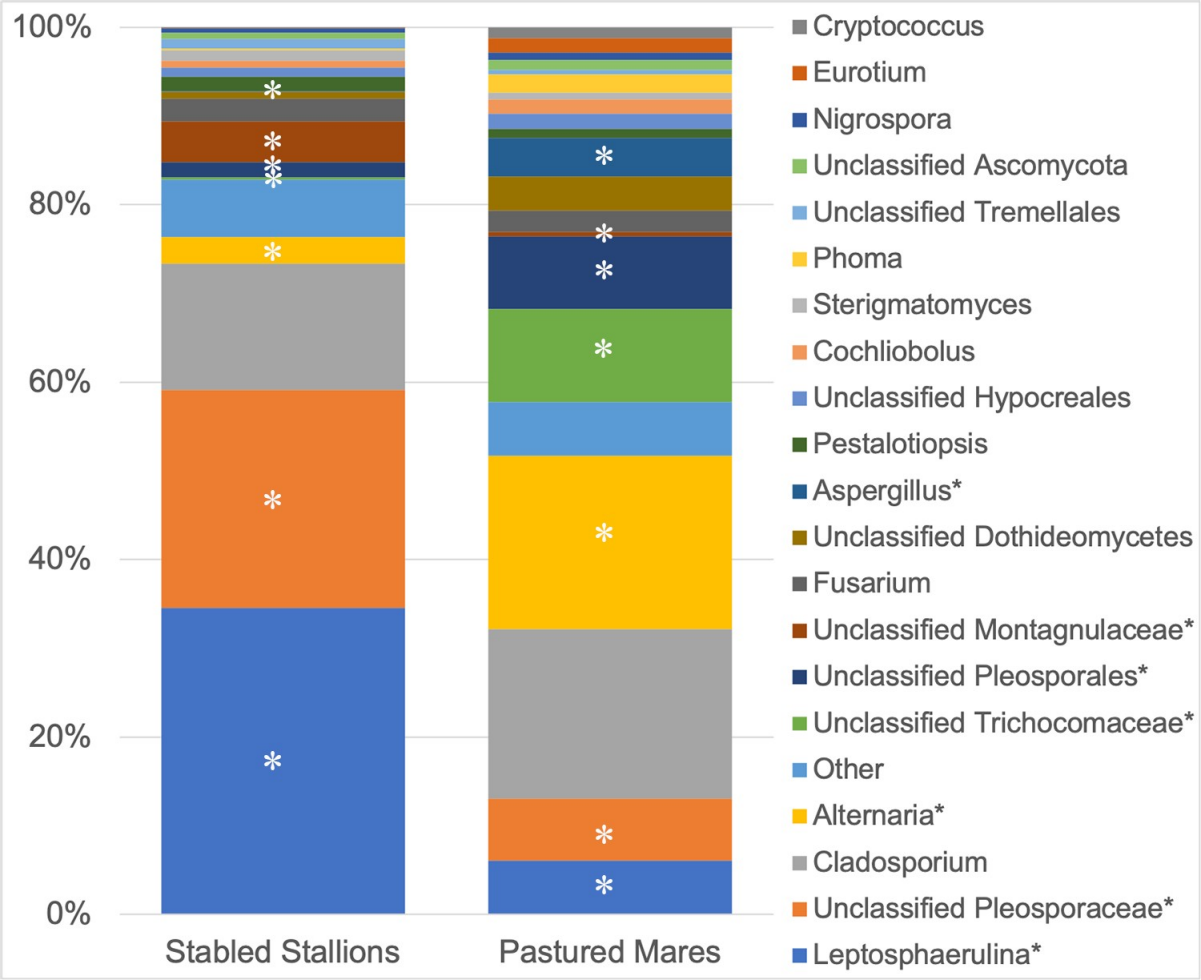
Composition of the ocular surface mycobiome in healthy horses separated by housing environment (stabled stallions vs. pastured mares). Relative abundance of taxa at the genus level. Note significant differences in abundance between groups (p < 0.05) annotated by (*).

Fungal community composition was then analyzed by housing environment. Table 2 compares the mean relative abundance of fungal genera sequenced from the eyes of stabled stallions and pastured mares. A Wilcoxon signed rank test was performed to identify which genera were significantly different between the two groups. Variance in the relative abundance of *Leptosphaerulina*, *unclassified Pleosporaceae*, *Alternaria*, *unclassified Pleosporales*, *unclassified Montagnulaceae*, *unclassified Trichocomaceae* and *Aspergillus* was observed between stallions stabled in an open-air pavilion and mares living on pasture (Table 2 and Fig 4). Specifically, *Alternaria* and *Aspergillus* spp. were significantly more abundant on the ocular surface of pastured mares compared to stabled stallions (Wilcoxon signed rank test; p = 0.002 and 0.005, respectively) (S1 and S2 Figs).

**Table 2:** Relative abundance of fungal taxa sequenced from the equine ocular surface of healthy horses in different housing environments.

TAXON Class	Mean Relative Abundance in Stabled Stallions (%)	Mean Relative Abundance in Pastured Mares (%)	P-value **
*Genus*			
**Dothideomycetes**			
Leptosphaerulina*	34.5	6.0	0.002
Unclassified Pleosporaceae*	24.6	7.0	0.005
*Cladosporium*	14.2	19.1	
Alternaria*	3.0	19.5	0.002
Unclassified Pleosporales*	1.7	8.2	0.044
Unclassified Montagnulaceae*	4.6	0.50	0.007
*Unclassified Dothideomycetes*	0.74	3.9	
*Cochliobolus*	0.8	1.6	
**Eurotiomycetes**			
Unclassified Trichocomaceae*	0.3	10.5	0.005
Aspergillus*	0.1	4.5	0.005
**Sordariomycetes**			
*Fusarium*	2.6	2.4	
*Pestalotiopsis*	1.7	1.0	
*Unclassified Hypocreales*	1.0	1.7	
**Agaricostilbomycetes**			
*Sterigmatomyces*	1.2	0.7	

*: Only p-values < 0.05 are shown.

**: P-values based on Wilcoxon signed rank test.

Mean relative percentages annotated to the level of genus are shown.

Similar to the variance in abundance reported in Table 2, LefSe analysis demonstrated that specific genera were altered depending on housing environment (Table 3).

**Table 3:** Linear discriminant analysis of fungal taxa in healthy horse eyes and their associations with housing environment.

Taxa (*Genus*)	LDA	Housing Environment
*Unclassified Dothioraceae*	3.378	Stable in open-air pavilion
*Unclassified Tremellales*	3.633	Stable in open-air pavilion
*Montagnulaceae*	4.322	Stable in open-air pavilion
*Unclassified Pleosporaceae*	4.945	Stable in open-air pavilion
*Leptosphaerulina*	5.145	Stable in open-air pavilion
*Cryptococcus*	3.777	Pasture
*Eurotium*	3.809	Pasture
*Phoma*	4.055	Pasture
*Aspergillus*	4.256	Pasture
*Epicoccum*	4.273	Pasture
*Cladosporium*	4.414	Pasture
*Unclassified Pleosporales*	4.495	Pasture
*Unclassified Trichocomaceae*	4.721	Pasture
*Alternaria*	4.928	Pasture

LDA scores > 3.0 are included.

## Discussion

This is the first report to describe the mycobiota of the equine ocular surface using next generation sequencing techniques. The present study confirms that the ocular surface of the healthy horse contains a greater diversity of mycobiota than suggested by previous culture-based studies. All 24 eyes sampled contained fungi from at least 5 genera at > 1% relative abundance (Table 1). The most relatively abundant genera included *Leptosphaerulina* (22.7%), *unclassified Pleosporaceae* (17.3%), *Cladosporium* (16.2%) and *Alternaria* (9.8%). These findings are similar to the previously described feline and canine conjunctival mycobiome where *Cladosporium* and *Alternaria* were prevalent; however, *Sporobolomyces* and *Malessezia* were identified in cats and dogs but not in horses at greater than 1% abundance [32, 34]. It is possible different genera were detected due to variations within the methods; however, this may also represent differences in core ocular mycobiomes between species and/or environments.

Previous culture-based studies have commonly identified numerous fungal organisms from the equine ocular surface including *Aspergillus*, *Cladosporium*, *Fusarium*, *Alternaria*, *Penicillium* and *Scopulariopsis* spp. [1, 8, 10–12]. While NGS detected *Cladosporium* and *Alternaria* with moderate relative abundance (16.2% and 9.8%, respectively), other genera such as *Fusarium* and *Aspergillus* only represented 2.5% and 1.9% of fungal genera sequenced, respectively. Additionally, *Penicillium* and *Scopulariopsis* were not reported in the current study as these organisms were not sequenced at > 1% relative abundance. This discrepancy may be explained by inherent differences between the two techniques. Fungal culture is biased towards fast growing and easily culturable organisms [39]. Due to large variations in growth requirements, culture media, and growth rates among fungi, numerous genera do not grow well in controlled laboratory conditions and are overlooked in culture dependent studies [31]. NGS targets a specific region of the fungal genome allowing for the identification of all fungi. However, this technique does not allow for the assessment of viability, thus it is possible some of the fungi identified in the current study are transient non-viable spores rather than established organisms inhabiting the ocular surface [24]. Molecular sequencing has identified numerous other genera never previously thought to contribute to the equine ocular mycobiome. These include *Leptosphaerulina*, *unclassified Pleosporaceae*, *unclassified Pleosporales*, *unclassified Montagnulaceae* and *unclassified Trichocomaceae*. While these organisms were identified, more research is needed to understand their function in the environment and impact on the health of the equine ocular surface.

Within physician ophthalmology, there is growing evidence supporting the capability of NGS to identify a distinct and diverse core ocular surface mycobiome compared to previous culture-based techniques. Only 6–12.5% of conjunctival swabs from healthy individuals yielded positive fungal culture growth, whereas 40–100% of swabs from the same individuals detected multiple fungal genera through NGS techniques [30, 31, 40]. The following genera were detected in the majority of human eyes sampled: *Aspergillus*, *Setosphaeria*, *Malassezia*, *Haematonectria* and *Alternaria*, among others [30, 31, 40]. These findings support the presence of a possible core ocular surface mycobiome which is more diverse than previously identified through culture-based techniques [30, 31, 40]. Additionally, humans with ocular disease such as fungal keratitis exhibited alterations in diversity and abundance of their ocular surface mycobiome compared to healthy individuals, suggestive of dysbiosis [40, 41]. This information may assist physicians treating affected patients as well as further our understanding of these disease processes. Future studies in veterinary medicine should determine if similar microbiome shifts occur in the face of ocular disease.

Reports of the cutaneous mycobiome sampled from multiple regions of the body describe the presence of an abundant and diverse community of organisms [29, 32, 34]. Direct regional comparisons of the mycobiome in cats and dogs determined that mucosal surfaces, including the nostril and conjunctiva, were significantly less diverse than other external areas of the body with haired skin [32, 34]. The ocular surface is thought to have relatively low biomass compared to other external regions of the body. This could be due to several protective mechanisms in place such tearing, blinking, and antimicrobial properties of the tear film [42]. While less abundant and diverse than the mycobiome of other external surfaces, the current study identified several fungal genera associated with the healthy equine conjunctiva with greater diversity compared to conventional cultivable methods. In physician ophthalmology, the definition of a core microbiome would include only those organisms found in all samples [43]. Using this definition, our results suggest that *Cladosporium*, *Alternaria* and *unclassified Pleosporaceae* constitute the core fungal microbiome in horses. Further research is necessary to confirm these findings.

It has been widely theorized that the environment affects which species of fungi colonize the conjunctival sac of horses. Previous culture-based studies evaluating the prevalence of fungi in different environments state that stabled horses are more likely to have positive fungal growth as compared to pastured horses as a result of their increased contact with hay and dust [10, 12, 13, 18, 35]. The mycobiome composition in horses may also reflect variations in the bedding substrate of stables. Our results support an environmental influence on the resident mycobiome; however, pastured horses were found to have a more diverse population of fungi as opposed to stabled horses. A significant difference in both alpha and beta diversity measures between groups revealed mares living on pasture had greater species richness, abundance, and taxonomic diversity compared to stallions stabled in an open-air pavilion. Within the microbial community composition, *Leptosphaerulina*, *unclassified Pleosporaceae*, *Alternaria*, *unclassified Pleosporales*, *unclassified Montagnulaceae*, *unclassified Trichocomaceae* and *Aspergillus* had a significantly different relative abundance between the mares housed on pasture and the stallions housed indoors. There was a significantly increased abundance of *Alternaria* spp. and *Aspergillus* spp. in the mares living on pasture (S1 and S2 Figs). This is of clinical interest as these fungal species are often identified in clinical cases of fungal keratitis. This statistical significance is also supported by LefSe (Table 3). Comparison of results of the LefSe analysis and Wilcoxon signed rank test revealed that *Leptosphaerulina* and *unclassified Pleosporaceae* were more prevalent on the ocular surface of stabled stallions compared to pastured mares. Conversely, *Aspergillus*, *Alternaria*, *Cladosporium* and *unclassified Trichocomaceae* were more abundant among pastured mares compared to stabled stallions. This is in contrast to what was described in the recently defined equine bacterial ocular surface microbiome, where no differences between sex or environment were identified in the same population of horses [25]. This could be attributed to the fact that fungi are ubiquitous organisms that feed on organic matter. Horses exposed to a high density and variety of organic plant material combined with larger fluctuations in humidity and temperature are more likely to harbor a greater diversity of fungal organisms compared to horses living in a more controlled environment. Living on pasture may therefore increase the risk for fungal infection if trauma occurs to the ocular surface.

There are limitations to the study population which represents two relatively small and homogenous populations. Although a homogenous sampling of horses decreases confounding variables, our findings may not be an accurate representation of the general horse population. Another limitation we encountered is that housing environment coincided with sex, as all mares were housed on pasture and all stallions were stabled. In the absence of data with males and females in both housing environments, we cannot completely rule out the possibility of sex influencing our results. However, the differences noted between groups in our study were most likely influenced by the environment rather than sex. When the cutaneous microbiota of indoor and outdoor cats was compared, the taxa Ustilaginomycetes and Ustilaginales were found to have greater relative abundance in the outdoor population [33]. In physician ophthalmology, Shivaji et al. describes that alpha diversity was not affected by sex; however, beta diversity was significantly affected [30]. Future studies are needed to eliminate this variable to confirm these findings. Additionally, horses included in the present study lived in the same geographic location and were sampled once in the winter. Future studies should evaluate the mycobiota in other geographic areas as well as assess for the effect of seasonality and temporal variation on the ocular surface mycobiome.

Further limitations exist with the interpretation of mycobiome studies. When selecting a primer pair set, a bias is inherently introduced. For our study, we used ITS1 and ITS2 primers for direct comparison to previous published studies of the animal surface mycobiota [32, 34]. The nuclear ribosomal RNA internal transcribed spacer (ITS) region has been demonstrated to have the highest probability for correct identification for a wide range of fungi [43]. However, fungi are an immensely diverse kingdom, which creates difficulty when trying to identify organisms and therefore when selecting the appropriate primers. While the ITS region is considered most effective, there are flaws in its ability to identify certain genera of fungi, including *Alternaria*, *Aspergillus*, *Cladosporium* and *Penicillium* [44, 45]. Therefore, it is possible these taxa were underestimated in our study. More recent primers have been developed that are proven to have greater coverage of fungal taxa on human body surfaces [46], and should be tested for animal studies in the future. Additionally, choice in fungal database has an important impact on fungal identification of surface mycobiota. In our study we selected the Findley et al. 2013 database for direct comparison to previously published studies of animal surface mycobiota. This database was comparable to UNITE for evaluation of the canine ear mycobiota [47, 48]. Further investigation is warranted for selection of the most appropriate and curated database for identification of fungi from other animal surfaces, but this was not within the scope of this clinical study.

Other limitations include the use of relative abundance which does not reflect absolute quantities of organisms present within samples. Microbiome datasets are considered compositional, yet the majority of microbiome analyses, including those employed in this study, use non-compositional models [49]. Therefore, changes in proportion do not always reflect changes in the absolute abundance of an organism [49]. Lastly, low biomass samples, even with the rigorous use of negative sample controls, may allow for reagent contaminants to impact the interpretation and reliability of the results [50, 51]. When there is low availability of DNA within the samples, there is little competition for contaminating DNA to bind with reagents for amplification [50, 51]. Future studies should include negative sequencing control blanks for use during DNA extraction and amplification to eliminate contamination. Despite these limitations, next generation sequencing has provided a wealth of data on the equine mycobiome which can be used to develop future studies to assess how the mycobiome is affected in diseased states.

## Conclusion

This is the first study to characterize the core ocular surface mycobiome in equids using next generation sequencing techniques. We identified the most prevalent genera including *Leptosphaerulina*, *unclassified Pleosporaceae*, *Cladosporium* and *Alternaria*. Alpha and beta diversity revealed that the resident mycobiota varied between horses, and is most likely affected by the housing environment with *Aspergillus* and *Alternaria* spp. having greater abundance in horses housed on pasture. Future studies should confirm that the mycobiome is not affected by gender and assess the mycobiome over different timepoints to confirm stability.

## Supporting information

S1 Fig Box and whisker plot demonstrating a significant difference in relative abundance of *Alternaria* on the ocular surface of healthy horses separated by housing environment (stabled stallions vs. pastured mares). P-values determined by Wilcoxon signed rank test with significance level < 0.05.
(TIF)

S2 Fig Box and whisker plot demonstrating a significant difference in relative abundance of *Aspergillus* on the ocular surface of healthy horses separated by housing environment (stabled stallions vs. pastured mares). P-values determined by Wilcoxon signed rank test with significance level < 0.05.
(TIF)

S1 Table Summary of alpha diversity indices at a depth of 12,900 sequences per sample. *P-values determined by Wilcoxon signed rank test with significance level < 0.05.
(DOCX)
