# Supplementary figures and images for: Evaluation of the ocular surface mycobiota in clinically normal horses
Source: PLoS One. 2021 Feb 4;16(2):e0246537. doi: 10.1371/journal.pone.0246537 (PMC7861450; doi:10.1371/journal.pone.0246537)

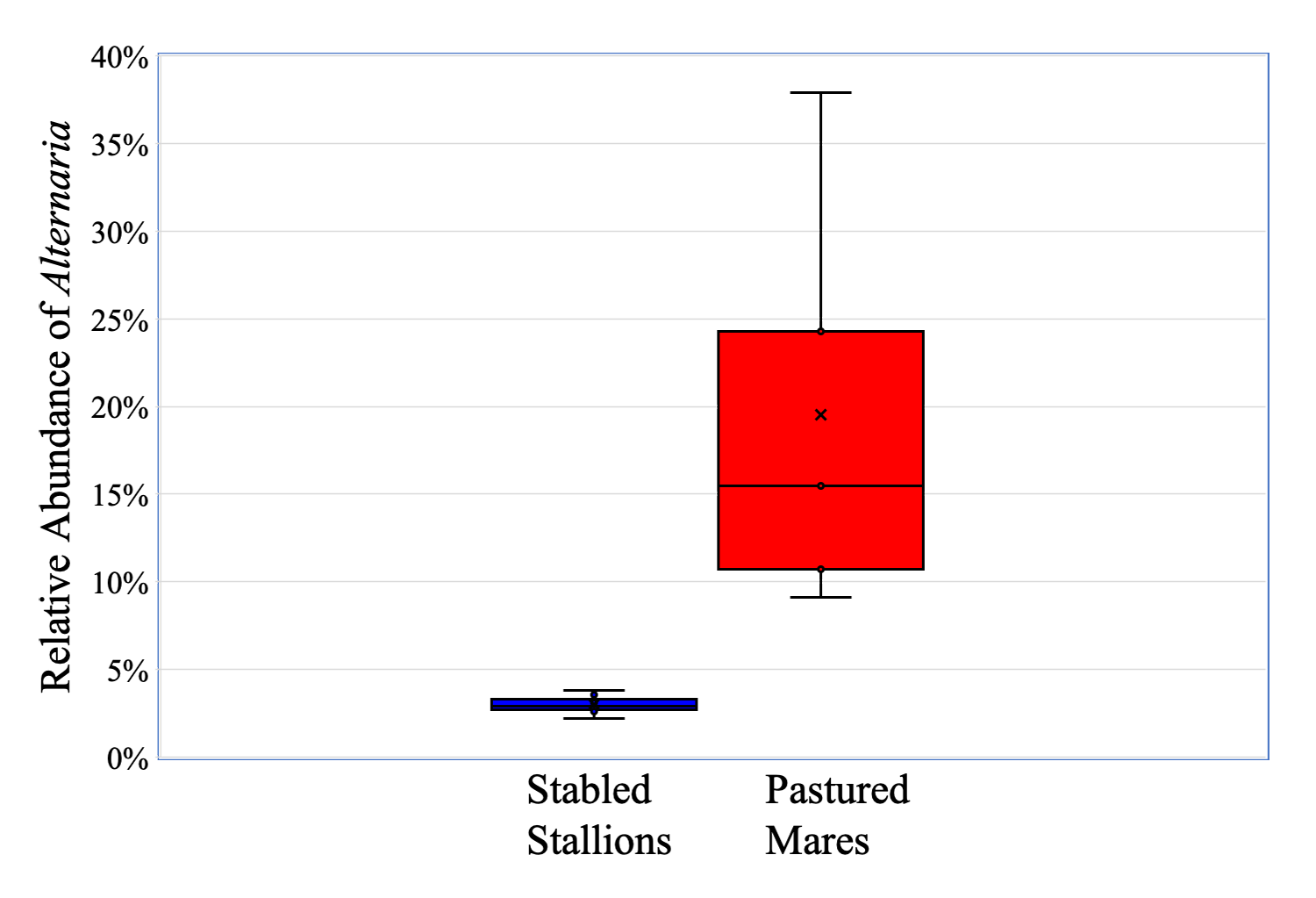

Supplement: S1 Fig — P-values determined by Wilcoxon signed rank test with significance level < 0.05. (TIF) [file pone.0246537.s001.tif]

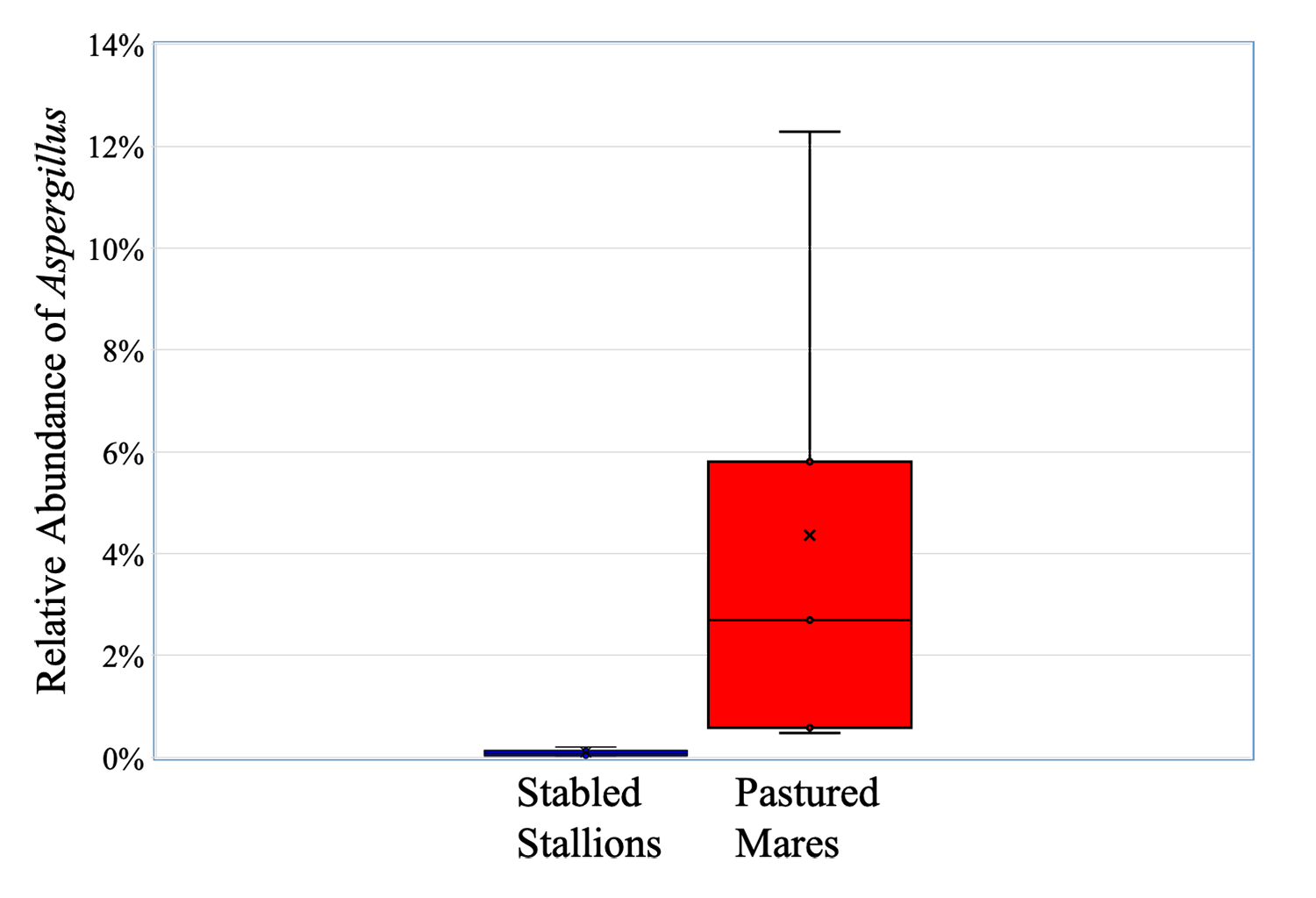

Supplement: S2 Fig — P-values determined by Wilcoxon signed rank test with significance level < 0.05. (TIF) [file pone.0246537.s002.tif]
