# Supplementary material for: Evaluation of the ocular surface mycobiota in clinically normal horses
Source: PLoS One. 2021 Feb 4;16(2):e0246537. doi: 10.1371/journal.pone.0246537 (PMC7861450; doi:10.1371/journal.pone.0246537)
Supplement: S1 Table — *P-values determined by Wilcoxon signed rank test with significance level < 0.05. (DOCX) [file pone.0246537.s003.docx]

**S1 Table. Summary of alpha diversity indices at a depth of 12,900 sequences per sample.**

|  | **Observed OTUs** | **Shannon** | **Chao1** |
| --- | --- | --- | --- |
| **Horses** | | | |
| Stallion 1 | 114.6 ± 8.3 | 3.1 ± 1.5 | 160.3 ± 11.6 |
| Stallion 2 | 99.1 ± 25.3 | 3.5 ± 1.6 | 142.8 ± 8.7 |
| Stallion 3 | 118.5 ± 13.3 | 4.0 ± 0.08 | 149.9 ± 7.1 |
| Stallion 4 | 123.4 ± 20.3 | 4.6 ± 0.06 | 153.4 ± 7.7 |
| Stallion 5 | 99.7 ± 27.8 | 3.5 ± 0.3 | 123.3 ± 47.4 |
| Stallion 6 | 86.6 ± 14.8 | 3.0 ± 0.3 | 117.6 ± 25.4 |
| Stallion 7 | 110.1 ± 12.9 | 3.1 ± 0.04 | 140.9 ± 13.9 |
| Mare 1 | 87.7 ± 12.1 | 3.2 ± 0.5 | 118.9 ± 17.9 |
| Mare 2 | 88.6 ± 5.6 | 3.0 ± 0.3 | 121.7 ± 2.9 |
| Mare 3 | 85.6 ± 12.6 | 3.1 ± 0.3 | 109.8 ± 16.9 |
| Mare 4 | 108.2 ± 6.9 | 3.1 ± 0.02 | 142.0 ± 9.8 |
| Mare 5 | 105.9 ± 3.8 | 3.3 ± 0.09 | 136.5 ± 8.6 |
| **^*^P-value** | 0.23 | 0.53 | 0.22 |
| **Environments** | | | |
| All Horses | 102.3 ± 17.0 | 3.4 ± 0.7 | 134.8 ± 20.7 |
| Stabled Stallions | 96.1 ± 13.3 | 3.1 ± 0.2 | 126.8 ± 16.6 |
| Pastured Mares | 111.1 ± 18.4 | 3.7 ± 0.9 | 145.9 ± 21.5 |
| **^*^P-value** | 0.016 | 0.022 | 0.006 |

Values represent averages with standard deviations. *P-values determined by Wilcoxon signed rank test with significance level < 0.05.
